# ESX1-dependent fractalkine mediates chemotaxis and *Mycobacterium tuberculosis* infection in humans^☆^

**DOI:** 10.1016/j.tube.2014.01.004

**Published:** 2014-05

**Authors:** Suzanne M. Hingley-Wilson, David Connell, Katrina Pollock, Tsungda Hsu, Elma Tchilian, Anny Sykes, Lisa Grass, Lee Potiphar, Samuel Bremang, Onn Min Kon, William R. Jacobs, Ajit Lalvani

**Affiliations:** a Tuberculosis Research Centre, Department of Respiratory Medicine, National Heart and Lung Institute, Imperial College London, London W2 1PG, United Kingdom; b Howard Hughes Medical Institute, Department of Microbiology and Immunology, Albert Einstein College of Medicine, New York, NY 10461, USA; c University of Oxford, Nuffield Department of Medicine, The Peter Medawar Building for Pathogen Research, South Parks Road, Oxford OX1 3SY, United Kingdom; d Tuberculosis Service, St Mary's Hospital, Imperial College Healthcare, National Health Service Trust, London W2 1PG, United Kingdom

**Keywords:** Tuberculosis, Fractalkine, Infection, Mycobacterium, ESX-1

## Abstract

*Mycobacterium tuberculosis*-induced cellular aggregation is essential for granuloma formation and may assist establishment and early spread of *M. tuberculosis* infection. The *M. tuberculosis* ESX1 mutant, which has a non-functional type VII secretion system, induced significantly less production of the host macrophage-derived chemokine fractalkine (CX3CL1). Upon infection of human macrophages ESX1-dependent fractalkine production mediated selective recruitment of CD11b+ monocytic cells and increased infection of neighbouring cells consistent with early local spread of infection. Fractalkine levels were raised *in vivo* at tuberculous disease sites in humans and were significantly associated with increased CD11b+ monocytic cellular recruitment and extent of granulomatous disease. These findings suggest a novel fractalkine-dependent ESX1-mediated mechanism in early tuberculous disease pathogenesis in humans. Modulation of *M. tuberculosis*-mediated fractalkine induction may represent a potential treatment option in the future, perhaps allowing us to switch off a key mechanism required by the pathogen to spread between cells.

## 1 Introduction

*Mycobacterium tuberculosis* (Mtb) remains the leading cause of bacterial mortality world-wide [1]. Little is known about the earliest events in human tuberculous infection during which the inhaled bacillus must spread from permissive cell to cell and establish its infective niche. Rapid availability of niche host cells early on (i.e. during the first 24–48 h) is essential for early establishment of infection and subsequent bacillary dissemination. The percentage of macrophages, representing the primary niche of the tubercle bacillus, in the human lungs is relatively low (ranging from 3 to 19%) with approximately 1 macrophage per 9 ml lung volume [2]. As such, a chemotactic call from the infected “Trojan horse” aggregating more of these naive cells could increase niche availability, cellular infection and bacterial dissemination.

It is increasingly recognised that chemotaxis-driven aggregation of macrophages is essential for establishment of the infectious niche and early bacillary spread in the *Mycobacterium marinum*-infected zebrafish [3]. This pioneering study reported how early granuloma formation (“macrophage infiltration and aggregation”) in the zebrafish resulted in increased bacterial growth and spread. Intriguingly, *M. marinum's* ability to establish infection and dissemination through early cellular aggregation was dependent on region of difference 1 (RD1), the most strongly virulence-associated genomic region of Mtb. Notably, the reduced cellular infiltration observed in transparent zebrafish embryos infected with the *M. marinum* RD1 mutant was due to reduced ESAT-6-mediated production of matrix metalloproteinase 9 (MMP-9) by epithelial cells which neighbour the infected macrophages [4]. The role of RD1, and hence ESX1, in early cellular aggregation and local spread of infection has not hitherto been tested in *M. tuberculosis* in humans.

Similarly to mutants of *M. marinum*, Mtb mutants without RD1 are highly attenuated yet unlike the zebrafish pathogen they have no defect in growth [5], [6] and thus represent a pure pathology-deficient phenotype or pat mutant [7]. Mtb region of difference 1 (RD1) represents the primary attenuating deletion of the vaccine strain Bacillus Calmette Guerin (BCG) [8] and spans a 9 gene region from *Rv3871-Rv3879*, central to which are the highly antigenic molecules early secreted antigen target-6 (ESAT-6) and culture filtrate protein-10 (CFP-10). Recently, RD1 was determined to be part of a novel type VII secretion system called ESX1 which is critical for virulence and was recently determined to be required for the survival of tubercle bacilli in the primate lung [9]. A number of virulence mechanisms have been attributed to ESX1 including phagosomal maturation arrest [10], cytosolic bacterial translocation [11] and host cell necrosis in cells 72 h post-infection [5], [12], [13], although it is unknown which of these is the most important in human tuberculosis *in vivo*.

The early virulence mechanisms utilised by the tubercle bacillus to obtain and set up its infective niche are likely pivotal in determining the outcome of *M. tuberculosis* exposure and infection and therefore important to elucidate. We hypothesised that ESX1 may play a similarly crucial role in early monocyte recruitment and bacterial dissemination in humans to that observed in the zebrafish. Using an *M. tuberculosis* mutant and human cells we assessed the role of ESX1 in *M. tuberculosis*-induced chemokine-mediated cellular aggregation and bacillary dissemination.

## 2 Materials and methods

### 2.1 Ethics statement

All samples were taken with approval from St. Mary's REC for 5 years (REC Number: 07/H0712/85) and with written informed consent. All animal work was carried out in accordance with the UK Animal (Scientific Procedures) Act 1986 and was approved by the animal use ethical committee of Oxford University.

### 2.2 Mtb growth

Mtb H37Rv was grown in Category III conditions on Middlebrook 7H9 (Becton Dickinson) medium plus 10% Middlebrook OADC (oleic acid, albumin, dextrose and catalase; Becton Dickinson) supplement, 0.5% glycerol (Sigma Aldrich) and 0.05% Tween 80 (Sigma Aldrich) at 37 °C at 100 rpm until they reached mid log phase when aliquots were frozen for use in 10% glycerol and plated out for colony forming units (CFU) on Middlebrook 7H10 agar plates plus 10% OADC and 0.5% glycerol at 37 °C for 2–3 weeks.

### 2.3 Infection experiments

Human THP-1 cells (a monocyte-derived cell line; American Type Culture Collection: ATCC), human A549 alveolar epithelial cells, murine J774 macrophage cells (ATCC), primary human alveolar epithelial cells (ATCC) or primary MDMs (isolated as in [5]) were infected at a multiplicity of infection (M.O.I) of 10:1, as described in detail in^5^. THP-1 cells were pre-treated with 20 ng/ml phorbol 12-myristate 13-acetate (PMA, Sigma Aldrich) for 18 h to stimulate adherence prior to infection or activation. In brief, cells were seeded in 12-well plates, or chamber slides (VWR) to determine successful intracellular infection, at 5 × 10^5^ cells/ml, and, after infection with bacilli for 2 h, any extracellular bacilli were washed off with warm RPMI and the media replaced. For supernatant analysis, the supernatant was removed and filtered using an ultra-free centrifugal filter device 0.22u (2 ml; Millipore) prior to removal from the CL3 suite. The cells were harvested for counting or flow cytometry by scraping. Trypan blue (Sigma) was used to determine viability, except in the flow cytometry where a viability stain was used (see below). To plate for extracellular CFUs a dilution series was prepared and plated out as above and for intracellular bacilli 0.1% Triton X-100 was used to lyse the cells prior to diluting and plating out. The anti-human CX3CL1/fractalkine antibody (R and D systems) was added at 1 μg/ml, the recombinant full length human CX3CL1/fractalkine (R and D systems) at 100 ng/ml, lipopolysaccharide at 10 ng/ml and piceatannol at 50 μM (Sigma Aldrich) for the stated time. For direct observation of the infected monolayers, coverslips were added to the well prior to the MDMs and staining was carried out using the TB cold stain kit (BDH).

### 2.4 Flow cytometry

Washed cells were stained with aqua LIVE⁄DEAD® Fixable Dead Cell Stain Kit, (Invitrogen), CD3-Pe-Cy7 (e-bioscience), CD11b-APC-Cy7 (BD bioscience) and FITC anti-human CX3CR1 (Cambridge Bioscience) at 1:100 dilutions for 30 min. Unstained controls were used to set voltages, single-stained controls for compensation and fluorescence minus one (FMO) controls for gate setting during analysis. The samples were run on a Becton Dickinson LSR Fortessa and analysed using FlowJo 9.3 (^©^TreeStar,Inc). Events were gated on live singlets then analysed for cell surface marker expression. Cells infected with Mtb H37Rv-FITC were stained with aqua LIVE⁄DEAD^®^ Fixable Dead Cell Stain Kit (Invitrogen) and then acquired on Becton Dickinson LSR Fortessa before analysis with FlowJo 9.3. The murine lung flow cytometry panel was composed of annexin-V/propidium iodide (AbCam), CD11b-APC-Cy7.7 (Becton Dickinson) and CD3-Pacblue (Cambridge Bioscience). For the single stained controls anti-rat/hamster murine beads were used (Invitrogen) and an unstained and FMO controls were also carried out. The samples were run on a Beckman Coulter CyAn ADP and live singlet cells were gated and analysed using Flowjo 9.3.

### 2.5 Murine experiments

Balb/C mice were infected intra-nasally with 300 CFU Mtb H37Rv or Mtb H37Rv ΔESX1. Mice (n3 per time point plus 1 naive uninfected control) were sacrificed at days 1, 3, 7, 14 and 28 post-infection. At each time point serum samples were taken and the lungs were resuspended in 2 mls of DMEM medium (Sigma Aldrich) plus 1.4 mg/ml collagenase (Sigma Aldrich) and 60 μg/ml DNase I (Sigma Aldrich). Following 45 min incubation at 37 °C the lungs were homogenised and the samples were centrifuged. The supernatant was saved as lung lysate and the lymphocytes were isolated from the cell pellet using Lympholyte M (CedarLane) and then processed for flow cytometry. At day 1 and day 28 post-infection one lung per mouse was homogenised and plated out for CFU as noted above in Mtb growth.

### 2.6 Modified trans-well assay

MDMs, matured for 6 days at 5 × 10^5^ cells per well, were infected with Mtb as above and 5 × 10^5^ PBMCs added in 12-well millicell hanging culture inserts with 5μ filters, which would allow both monocytes and lymphocytes to pass through (Merck Millipore). Following 24 h infection (∼maximal fractalkine production) the inserts were removed and the cells were harvested by centrifuging the supernatant and by scraping for adherent cells prior to counting in a haemocytometer and flow cytometry staining.

### 2.7 Multiplex cytokine assays

The 12-plex human cytokine array MPXHCYTO-60K-12 (Merck Millipore), human fractalkine single-plex MPXHCYTO-60K-01 (Merck Millipore), murine single-plex MPXMCYP2-73K-01(Merck Millipore) and matrix metalloproteinase panel 1 HMMP1-55K-03 (Merck Millipore) were carried out as per the manufacturer's instructions.

### 2.8 Necrosis assay

LDH release was quantified at specified time points by using the cytotoxicity detection kit (Roche Diagnostics), according to the manufacturer's instructions. The maximum and background controls used were 0.1% Triton X-100-treated cells (maximum) and uninfected cells (background). Values were calculated by using the following equation: % LDH release: (sample-background)/(maximum-background) × 100. The necrosis positive control was 0.1% Triton X-100 (Sigma).

### 2.9 Bronchoalveolar lavage (BAL) samples

BAL samples were obtained from the Tuberculosis Service at St Marys hospital, transferred to CL3 facilities and following passage through a 70μ cell strainer (VWR) and centrifugation the cells were pelleted and frozen in foetal calf serum plus 10% DMSO (Sigma Aldrich) at −80 °C until use in flow cytometry. The supernatant or BAL filtrate was filtered through a 0.2μ filter and stored at −20 °C until use. BAL samples were also acquired from healthy and asthmatic individuals too.

## 3 Results

### 3.1 *Mtb* infection of THP-1 macrophages results in the production of fractalkine which is mediated by the virulence-associated ESX1

We previously constructed a Mtb H37Rv mutant in the RD1 region *Rv3871-Rv3879* which has a defective ESX1 secretion system [5]. We infected the adherent THP-1 cells at a multiplicity of infection (M.O.I) of 10:1 with the wild-type and mutant strains and took supernatants at 24 h and 48 h post-infection (since the H37Rv ESX1 mutant exhibits a defect in cellular necrosis at 72 h post infection [5]). Along with the mutant and wild-type, infection was also carried out with a complemented mutant [5] and controls included uninfected and LPS-treated adherent human cells.

**Figure 1:**
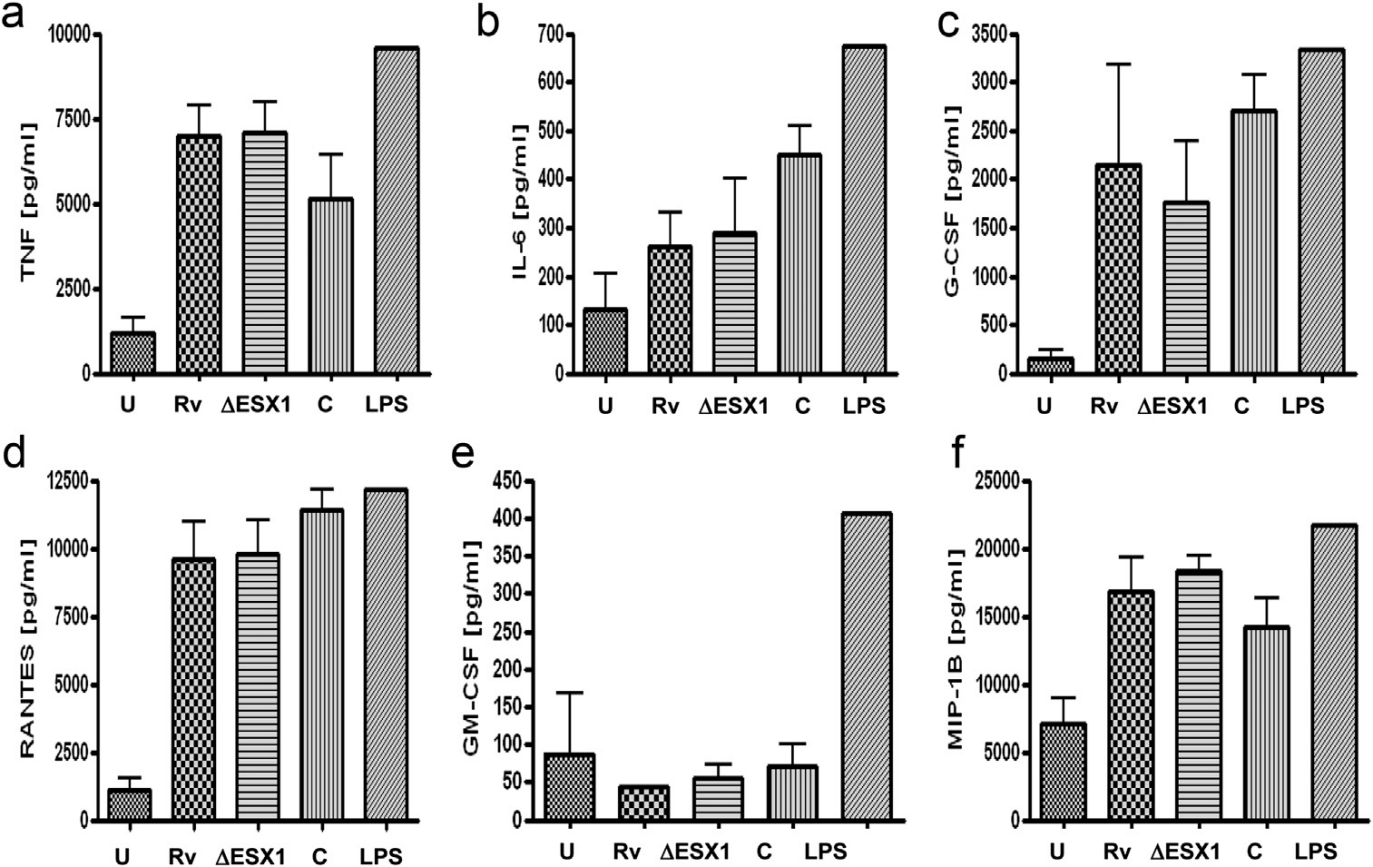
**Mtb H37Rv induces the production of key macrophage cytokines in a non-ESX1 dependent manner (24 h post-infection)** Multiplex cytokine array for a) tumour necrosis factor alpha (TNF-a), b) interleukin 6 (IL-6), c) granulocyte colony stimulating factor (G-CSF), d) granulocyte macrophage colony stimulating factor (GM-CSF), e) macrophage inflammatory protein 1beta (MIP-1B) and f) regulated on activation normal T cell expressed and secreted (RANTES). Samples analysed were PMA-treated THP-1 cells uninfected (U), H37Rv infected (Rv), H37Rv ΔESX1 infected (ΔESX1), H37Rv ΔESX1 complemented (C) and LPS (10 ng/ml) stimulated for 24 h hours at an M.O.I. of 10:1. Pg = pg/ml. This is representative of triplicate experiments and the bars shown are standard deviations.

**Figure 2:**
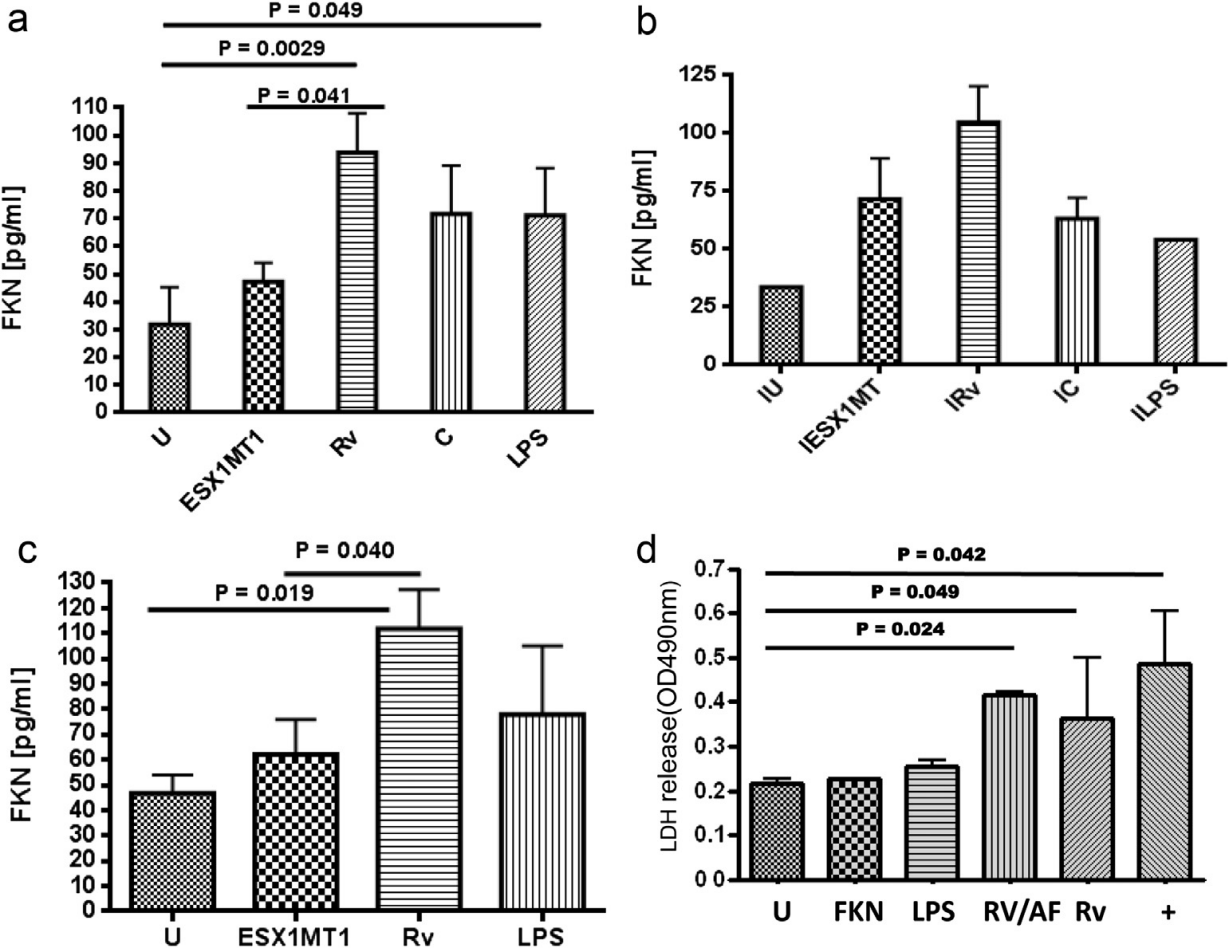
**The production of fractalkine from THP-1 cells and human macrophages at 24 h post-infection is ESX1-dependent and does not cause necrosis**. a) Fractalkine production as measured by a multiplex cytokine assay from THP-1 cells activated overnight with PMA and infected with an M.O.I. of 10:1 with H37Rv (Rv), H37Rv ΔESX1 (ΔESX1), complemented H37Rv ΔESX1 (C), untreated (U) or LPS treated (LPS, 10 ng/ml). b) Fractalkine production from THP-1 cells plus overnight IFN-γ (I) stimulation and infected as a. c) Fractalkine production from monocyte-derived macrophages from healthy controls and infected as a. d) Host cell necrosis, as measured by lactate dehydrogenase release, from human monocyte-derived macrophages at 72 h post-infection at an M.O.I. of 10:1. U = uninfected, FKN = treated with FKN, LPS = LPS treatment at 10 ng/ml, RV = H37Rv, RV/AF = Rv plus anti-fractalkine, + = Triton X-100 treated. Each experiment has been repeated three times, from three different healthy donors if primary cells. Statistical analyses were carried out using the Mann–Whitney non-parametric test.

Secretion of all of the innate cytokines measured in response to infection with Mtb was similar to that with infection with the Mtb ΔESX1 mutant (Figure 1a–f), with the exception of the chemokine fractalkine (Figure 2a, *P* = 0.041). Therefore, uniquely among the panel of chemokines and cytokines evaluated, fractalkine induction at 24 h post-infection in Mtb-infected THP-1 cells is ESX1-mediated. This difference was also observed at 48 h post-infection (data not shown) but in both cases was only observed in resting macrophages, the suggested preferred interstitial niche of the tubercle bacillus [14] and was not seen in cells activated by the Th1 cytokine interferon-γ (IFN-γ; Figure 2b).

### 3.2 Fractalkine production is ESX1-mediated in primary human MDMs and does not induce cellular necrosis

We also determined that fractalkine production was ESX1-dependent in primary human MDMs (Figure 2c, *P* = 0.040). In contrast, there was no production of fractalkine noted in mruine macrophages. The ESX1 region of Mtb is also associated with increased cellular necrosis and cellular egress at 72 h post-infection [5] so we assessed whether fractalkine plays a role in cellular necrosis at this time point using an anti-fractalkine monoclonal antibody, recombinant fractalkine and Triton-X 100 induction of necrosis as a positive control. Infection with Mtb H37Rv resulted in the induction of necrosis similar to the Triton-X 100-treated positive control but this was not reduced by the addition of anti-fractalkine (Figure 2d). Moreover, the addition of fractalkine itself did not increase necrosis over the basal uninfected level confirming that fractalkine does not induce macrophage necrosis in this system.

### 3.3 Matrix metalloproteinase 1, 2, 7, 9 and 10 production from Mtb-infected human macrophages and epithelial cells is not ESX1-mediated

**Figure 3:**
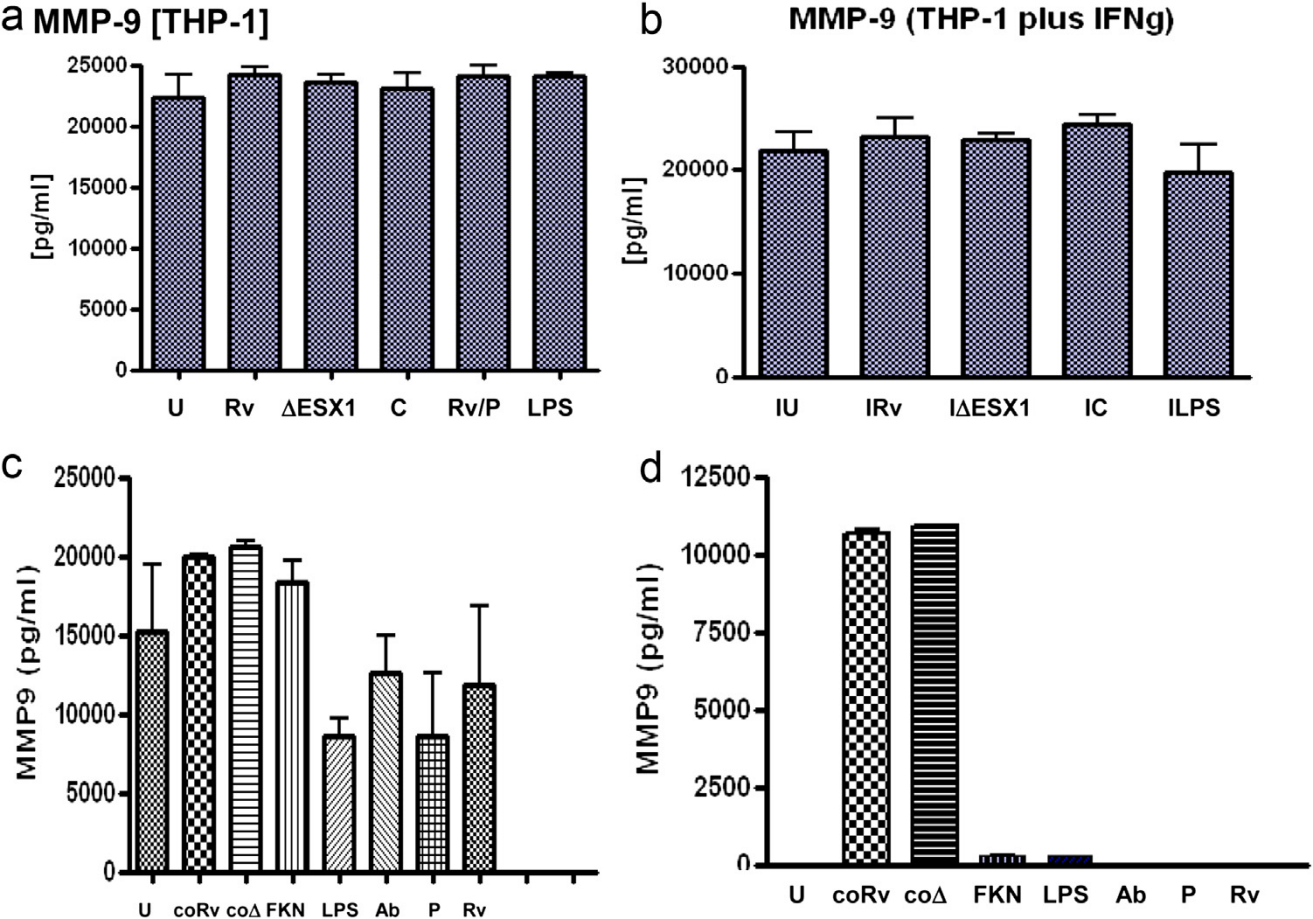
**MMP-9 is produced by human macrophages at 24 h post-infection, but is not ESX1-mediated**. Cells were infected at an M.O.I. of 10:1 or exposed to conditioned medium for 24 h. U = uninfected, Rv = Mtb H37Rv, ΔESX1 = Mtb ESX1 mutant, C = complemented ESX1 mutant, P = Mtb plus piceatannol, Ab = Mtb plus anti-fractalkine antibody, coRv = conditioned medium from Rv infected cells, coΔ = conditioned medium from ESX-1 infected cells, LPS = 1 ng/ml a) THP-1 cells, b) THP-1 cells plus overnight 10 U IFN-γ, c)MDMs, d) A549 alveolar epithelial cells. Data is representative of two replicates and 2 independent experiments and shows standard error bars.

Elucidation of the fundamental role of *M. marinum*-induced ESX-1-dependent MMP-9 secretion in zebrafish embryos [4] after we generated the above data prompted us to investigate ESX1-dependence of MMP-9 secretion in our human system. We assessed production of MMP-9 in THP-1 cells, both with and without IFN-γ, and in human MDMs and found no difference between Mtb H37Rv and the Mtb ΔESX1 mutant. This was also the case with cells activated with conditioned media from infected macrophages (Figure 3a–c). Although we observed substantial production of MMP9 in human alveolar epithelial cells (A549 cell line; Figure 3d) there was no ESX-1 mediated difference. The zebrafish immune system differs substantially from the human, so we studied several other MMPs (1, 2, 7 and 10) to see if an alternative MMP might be acting in the place of the zebrafish MMP9 and there was no observable ESX1-mediated difference in the MMPs 1, 2 7 or 10 (Supplementary Figure 1).

### 3.4 Mtb ESX1-mediated fractalkine production results in Syk-dependent monocytic cell recruitment and *M. tuberculosis* niche expansion in humans

**Figure 4:**
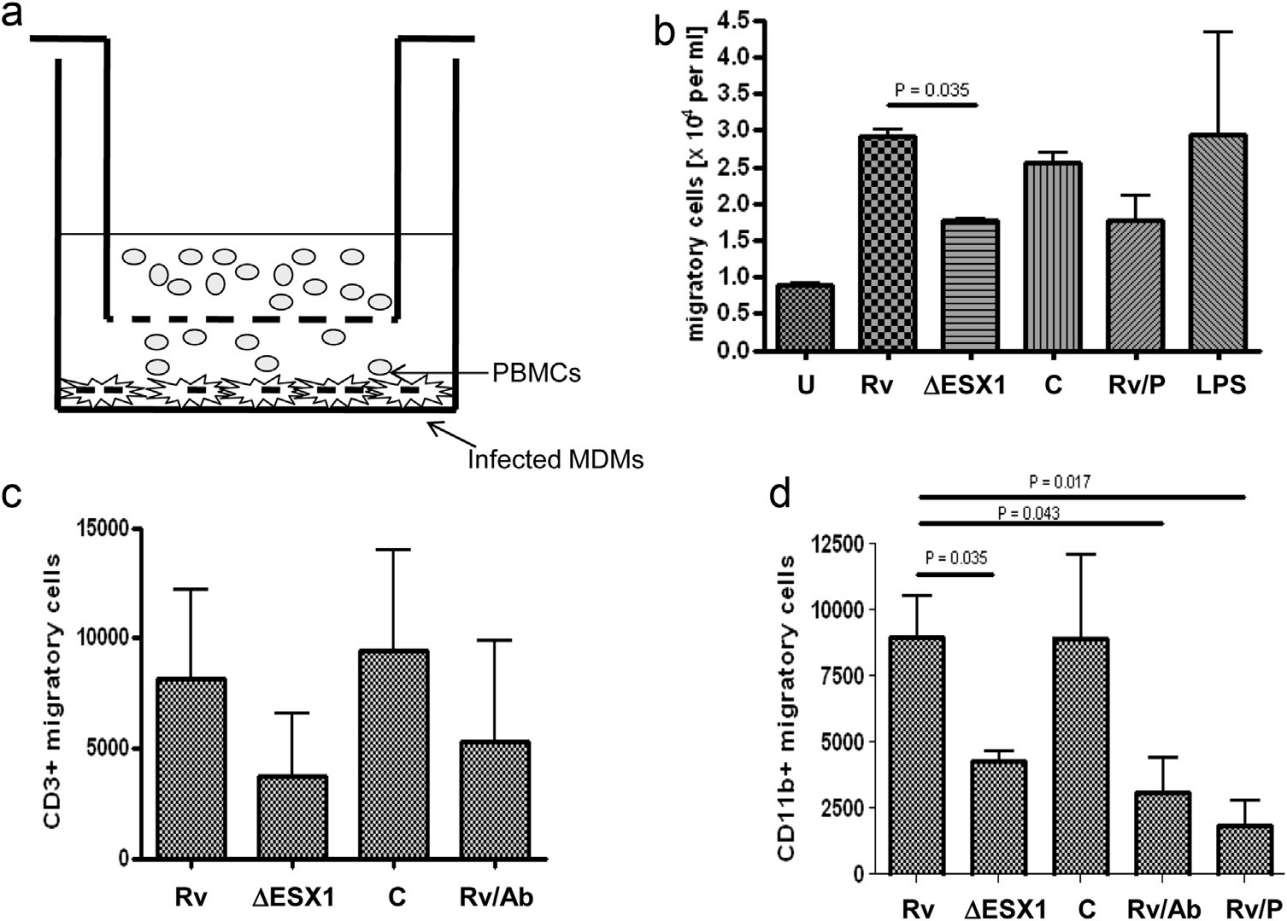
**ESX1-mediated fractalkine production results in migration of CD11b+ cells**. a) Modified trans-well assay set up shown per well with transwell insert and with infected MDMs and PBMCs, b) migrating cells as measured per ml on a haemocytometer after trypan blue staining, c) flow cytometric determination of CD3+ migrating cells and d) flow cytometric determination of CD11b+ migratory cells. Each graph is representative of three independent experiments with standard error bars. Statistical analyses were carried out using a Mann Whitney statistical analysis for figures b–d.

Fractalkine's primary function is as a chemoattractant to both monocytic and lymphocytic cells. Accordingly we set up a modified transwell assay (see Figure 4a) in order to test for Mtb-induced ESX1-dependent cellular migration. Direct observational counting in a haemocytometer on blinded samples revealed a reduction in cellular migration in the Mtb ΔESX1-infected transwell experiment in all three healthy donors. This difference was significantly different to wild-type-infected cells and was restored by complementation of the ESX1 mutation (Figure 4b). The cytoplasmic tyrosine kinase Syk is required for monocyte/macrophage chemotaxis to fractalkine [15] and addition of the Syk inhibitor picetannol resulted in a similar migratory reduction to that observed with Mtb ΔESX1 confirming that Syk-dependent ESX1-mediated fractalkine production results in cellular migration towards Mtb-infected cells.

We then determined the nature of these migratory cells using flow cytometry. We stained for dead cells using a live/dead discrimination kit then stained with the fluorochrome-conjugated antibodies, CD3-Pe-Cy7 and CD11b-APC-Cy7. CD3 is a pan-T cell marker and CD11b is an integrin also known as Mac-1 often used as a pan monocyte-macrophage marker and used in murine studies for macrophage depletion [16]. While the CD3+ cell number exhibited no difference (Figure 4c), the number of CD11b+ cells was significantly reduced in the Mtb ΔESX1-infected transwell assay when compared to the Mtb wild-type-infected cells (Figure 4d). A similar reduction was seen in CD11b+ cell migration when the wild-type infected cells were treated with an anti-fractalkine antibody or the Syk inhibitor picetannol, but not with an anti-TNF antibody (data not shown). Thus, Mtb ESX1-mediated fractalkine production results in enhanced recruitment of CD11b+ monocytic cells, the niche for the tubercle bacillus, but not in CD3+ T cells, many subtypes of which are implicated in protection against tuberculosis.

### 3.5 Increased monocytic cellular recruitment results in increased percentage infection and cellular spread

**Figure 5:**
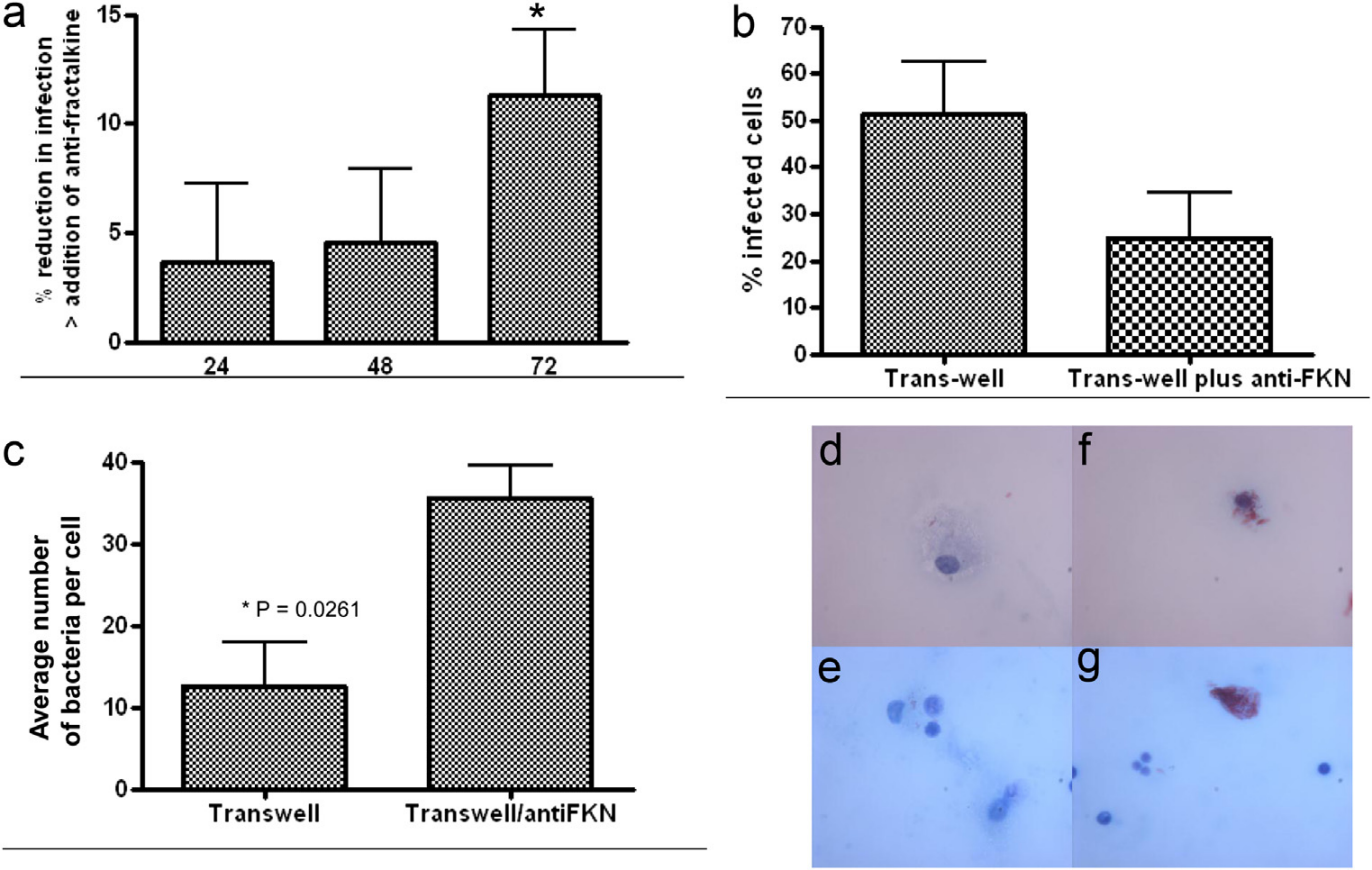
**The addition of anti-fractalkine results in reduced rates of cellular infection and increased bacillary numbers per cell**. a) % reduction in infection of Mtb H37Rv-FITC infected cells after addition of the anti-fractalkine antibody and as measured by flow cytometry, b) % cells infected from infected monolayers stained with carbol fuschein and malachite green counter-stained. Cells were counted using oil immersion light microscopy at a magnification of × 100 and from at least 10 different fields, c) enumeration of intracellular bacteria from infected monolayers as 5b. Representative fields are shown in Figure 5d–g, where 5f and g show the anti-fractalkine treated wells and 5d and e the untreated wells. Figures each represent three independent experiments from different donors. Statistical analyses were carried out using a Dunns multiple comparison test for 5a and b and a student's *t*-test for 5c.(For interpretation of the references to colour in this figure legend, the reader is referred to the web version of this article.)

The Mtb ESX1 mutant does not exhibit a growth defect in human cells or *in vivo* in the murine model [5] and this was also determined to be the case in the transwell system by plating out colony forming units (CFU) from 0, 24, 48 and 72 h post-infection plus and minus anti-fractalkine antibody (data not shown). However, we hypothesised that the increased recruitment of niche cells for the bacilli would result in an increase in the percentage infection rate of the cells early after infection. Therefore, fluorescently labelled wild-type bacteria (FITC-labelled Mtb H37Rv) were used to infect MDMs in the modified transwell experiment with and without anti-fractalkine antibody and the numbers of infected cells determined using flow cytometry in the FITC channel. The percentage infected cells were significantly reduced upon treatment with anti-fractalkine, which reduces CD11b+ monocytic cellular migration (Figure 5a, *P* < 0.05).

Direct observation, again blinded, of the infected monolayers was also carried out following Ziehl-Neelsen staining. This confirmed the above finding, showing reduced infection levels in the anti-fractalkine treated wells 72 h post-infection (Figure 5b). This also enabled us to count the number of bacilli per infected cell (Figure 5c) and there were significantly higher (*P* = 0.026) numbers of intracellular bacteria associated with the anti-fractalkine treated cells (mean = 35) than with the control (mean = 12) at this time point. Representative fields are shown in Figure 5d–g, where 5f and g show the anti-fractalkine treated wells and 5d and e the untreated wells. Blocking fractalkine thus results in higher numbers of bacilli per cell and fewer infected cells, indicating that fractalkine-mediated monocyte influx early in infection aids in bacillary spread. ESX1-mediated fractalkine production from infected macrophages thus results in increased influx and infection of monocytic cells and promotes bacillary dissemination.

### 3.6 Increased fractalkine levels are associated with granulomatous disease and increased CD11b+ monocytic cellular infiltration at disease sites in humans

**Figure 6:**
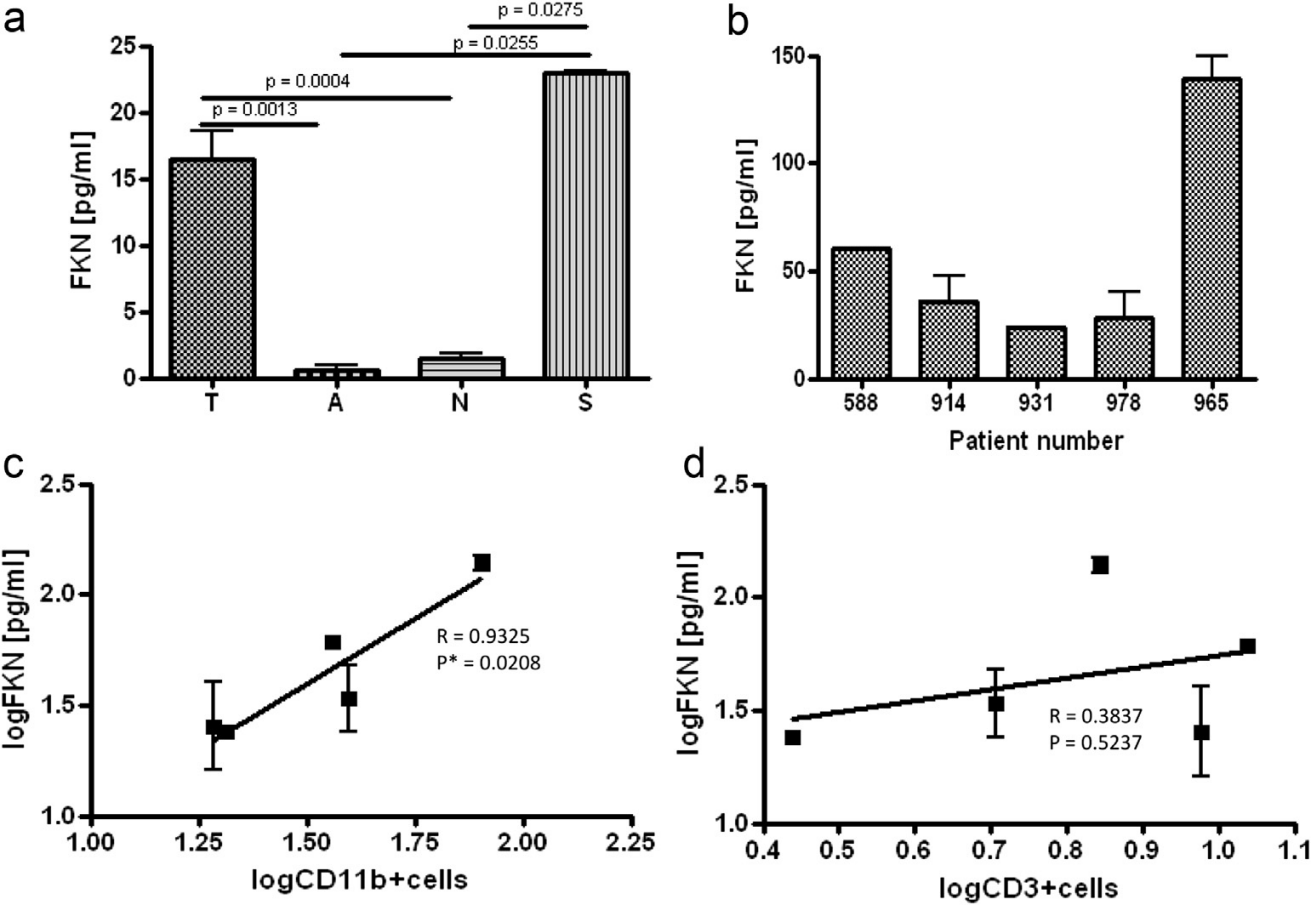
**Increased FKN levels are associated with granulomatous disease in humans and correlate with increased CD11b+ and not CD3+ cellular infiltration in the human lung**. a) FKN levels as measured by multiplex cytokine assay in BAL samples from TB patients (n13), asthmatic (n3), healthy controls (n3) and sarcoid patients (n3), b) FKN levels in BAL filtrate from patients with active tuberculosis (duplicate samples), c) log number of CD11b+ cells present in BAL samples from patients with active tuberculosis versus log FKN levels and d) log number CD3b+ cells present in BAL samples from patients with active tuberculosis versus log FKN levels. Statistical analyses were carried out using a Mann–Whitney statistical analysis for 6a and Pearsons rank test for 6c and d.

We sought to determine the involvement of fractalkine in granulomatous disease, employing sarcoidosis as an example of a non-TB disease associated with the formation of granulomas in the lung. Therefore, we obtained bronchoalveolar lavage (BAL) fluid from patients with TB (n13), sarcoidosis (n3), asthma (n3) and normal healthy (n3) controls, filtered the supernatants and measured fractalkine levels using a multiplex cytokine assay. The BAL fluid from TB patients contained significantly higher levels of fractalkine than the inflammatory but non-granulomatous lung disease asthma (*P* = 0.001) and the healthy controls (*P* = 0.004, Figure 6a). Sarcoidosis also exhibited significantly increased levels of fractalkine compared to asthma (*P* = 0.026) and healthy controls (0.028) suggesting an association with granulomatous disease.

In 5 patients with active cavitatory pulmonary tuberculosis with consolidation, we enumerated the number of CD11b+ and CD3+ cells by flow-cytometry in BAL and correlated these with fractalkine levels (Figure 6b) in filtrates of the same BAL samples. The levels of fractalkine at the site of disease clearly correlated with the number of CD11b+ cells (Figure 6c, *P* = 0.021), but not CD3+ cells (Figure 6d, *P* = 0.383), thus suggesting that increased fractalkine in the lung is significantly associated with increased monocytic cell numbers and niche expansion. This increase in available niche cells would be expected to result in increased cellular infection and egress, potentially associated with more disseminated disease in the lung.

### 3.7 Mtb-mediated fractalkine production and CD11b+ cellular migration was not observed in the murine model of infection

A major limitation of the murine model of infection with Mtb is the lack of granuloma formation. We intra-nasally infected Balb/C mice with the Mtb ΔESX1 mutant and wild-type strains. No ESX1-mediated differential fractalkine (generally called lymphotactin in the mouse with structural differences to the human homologue [17]) production was observed in the lung lysates (as had been seen previously in murine macrophages *in vitro*) and no differential infiltration of CD11b+ or CD3+ cells was observed between the mutant and wild-type at days 1, 3, 7, 14, 21 and 28 (data not shown). In addition, there was no difference noted in colony forming units as previously determined [5].

## 4 Discussion

The early events in TB infection are likely critical to determining whether the outcome favours the host or the pathogen. Following inhalation the bacilli must infect and then replicate within its primary niche cell; the macrophage. At this early stage the absence of further permissive niche cells will halt the spread of infection and conversely the influx of niche cells would enable the infection to spread. Here we show that the tubercle bacillus utilises the very cell-type evolved to destroy invading pathogens in order to establish and locally disseminate infection by signalling sibling-cells and increasing its permissive niche. Our findings suggest that the Mtb-mediated influx of uninfected niche cells allows the bacteria to infect new host cells, disseminate and to set up a foothold of infection within the human lung.

Elegant studies in the *M. marinum*-infected zebrafish model of infection describe how this early aggregation of macrophages, termed a granuloma, is utilised by the bacilli to facilitate bacterial growth and the spread of infection [3]. However, although the *M. marinum*-zebrafish model has fundamentally advanced our understanding of early Mtb infection, zebrafish do not have lungs and so whether or not ESX1-dependent cellular aggregation and infectious dissemination occurs in the lung is unknown. In addition, zebrafish embryos lack a fractalkine homologue and also lack T and B cells [18], so whether preferential recruitment of macrophages also occurs in models with both lymphoid and myeloid cells in humans needs to be determined. Using a non-growth deficient Mtb ESX1 mutant, unlike the *M. marinum* mutant, here we provide the first evidence of ESX1-dependent specific fractalkine-mediated chemotaxis of monocytic cells to infected macrophages in the presence of lymphocytes. We further demonstrate a relationship between monocyte infiltration and fractalkine levels in tuberculous human lung.

The chemokine fractalkine (CX3CL1) exists in a class of its own containing a unique C-X3-C motif and can exist in both membrane-bound and soluble forms, the latter of which has potent monocyte and T cell chemoattractant properties, higher than the chemokine RANTES [19]. Fractalkine is secreted by many cell types including macrophages and can exist as a membrane bound molecule only in endothelial cells due to the presence of a mucin-like stalk, which is cleaved from the soluble form [20]. Although fractalkine has been determined to be present at the site of disease in patients with pulmonary tuberculosis [21], [22] its role in human tuberculosis was hitherto unknown. The cognate receptor for fractalkine, CX3CR1, is necessary for migration and not adhesion and shown to be expressed by the majority of monocytes, NK cells and about 10–15% of T cells [23]. Interestingly, CX3CR1 is a requisite for rapid tissue invasion of resident monocytes at the site of infection in mice peritoneally infected with *Listeria monocytogenes* [24].

We identified ESX-1-dependent induction of the chemokine fractalkine from host macrophages. A modified trans-well migration assay revealed ESX-1-mediated FKN-dependent migration of CD11b+ (a pan-macrophage marker) cells, with no significant effect upon migration of CD3+ (pan-T cell marker) cells at this sample size. The recruitment of uninfected macrophages by fractalkine by the Mtb-infected macrophage resulted in productive infection, increasing the proportion of infected cells and thus bacillary dispersal and dissemination. The addition of anti-fractalkine resulted in fewer infected cells with higher bacterial loads.

We next assessed the role of fractalkine *in vivo*. In our murine experiments there was no fractalkine production and no ESX1-mediated difference in cellular recruitment of CD11b+ or CD3+ cells into the murine lung, suggesting this artificial model system which lacks granuloma formation and which, unlike the zebrafish-*M. marinum* model, is not a natural host/pathogen pair, is substantially different to human infection. *In vivo* in the human lung, fractalkine levels were elevated in BAL from TB patients compared to healthy controls and asthmatic controls and were moreover strongly correlated with the extent of monocytic cell infiltration. Fractalkine levels were also elevated in BAL from sarcoidosis patients, suggesting an association of fractalkine with pathogenesis of granulomatous disease, consistent with earlier observations in TB, sarcoidosis and Wegeners granulomatosis [22], [25].

The role of fractalkine in granulomatous disease clearly requires further investigation and, in the case of TB, it would be of interest to investigate ESX-1-mediated fractalkine-induction in a human *in vitro* mycobacterial granuloma model similar to that of Birkness et al. where the involvement of the cytokines IFN-γ and TNF-α was elegantly dissected [26]. Fractalkine itself acts as a potent polariser of the Th1 immune response and its role in the adaptive immune response to Mtb remains to be fully elucidated. Increased fractalkine has also been associated with factors which increase the risk of the development of TB disease and of disease reactivation including age [27] and HIV infection [28]. Further work is now required to correlate fractalkine levels with bacillary burden and patient outcomes including risk of disease progression and response to therapy.

The type VII secretion system ESX1 system has also been implicated in cellular necrosis leading to increased cellular egress later in infection [5] and fractalkine could represent an early stage recruiting permissive cells prior to their release by necrosis. Indeed, abrogating the effect of fractalkine resulted in reduced CD11b+ cellular recruitment and reduced percentage infection, without affecting cellular necrosis. The ESX1-secreted protein ESAT-6 was responsible for the MMP9 production from neighbouring epithelial cells in *M. marinum*-infected zebrafish embryos [5], [29]. The complex secretory co-dependence of this system makes the exact role of each of the ESX1-secreted proteins difficult to dissect and this work is currently ongoing.

In conclusion, Mtb ESX1-fractalkine-mediated CD11b+ cellular migration may play a role in establishment of a foothold in initial infection, maintenance and spread of bacilli through niche expansion. Our data moreover suggest that the fractalkine/fractalkine receptor axis could represent an attractive target for novel immunomodulatory interventions for treatment or prevention of tuberculosis infection. A human fractalkine analogue which inhibits CD11b+ chemotaxis in human MDM's has recently been described [30] and our findings support the clinical evaluation of this as a potential novel strategy to reduce the spread of the tubercle bacilli *in vivo*.

## Ethical approval

All samples were taken with approval from St. Mary's REC for 5 years (REC Number: 07/H0712/85) and with written informed consent. All animal workwas carried out in accordance with the UK Animal (Scientific Procedures) Act 1986 and was approved by the animal use ethical committee of Oxford University.

## Financial support

This work was funded by the Wellcome Trust.

## Author contributions

S. M. H-W. and A.L. designed the experiments and wrote the paper. S. M .H-W carried out the experiments with the exception of the mouse experiments which were carried out by E.T., O.M.K, L.G., L.P and A.S. provided the BAL samples and D. C. and K. P. helped with their analysis. S. M. H-W., T. H. And W. R. J Jnr created the Mtb ESX1 mutant.

## Competing financial interests

The authors declare no competing financial interests.
