# Supplementary material for: ESX1-dependent fractalkine mediates chemotaxis and Mycobacterium tuberculosis infection in humans
Source: Tuberculosis (Edinb). 2014 May;94(3):262–70. doi: 10.1016/j.tube.2014.01.004 (PMC4066952; doi:10.1016/j.tube.2014.01.004)

Supplementary Figure 1 Legend

MMP-1, 2, 7 and 10 are produced by human monocyte-derived macrophages at 24h post-infection, but are not downregulated in ∆ESX1. Cells were infected at an M.O.I. of 10:1 or exposed to conditioned medium for 24 hours. a) MMP1, b) MMP2, c) MMP7 and d) MMP10 concentrations in pg/ml as determined by multiplex cytokine analysis. 1) uninfected, 2) CoH37Rv, 3) co∆ESX1, 4) Rv, 5) ∆ESX1, 6) H37Rv+Ab, 7) FKN and 8) LPS. Data is representative of two replicates and 3 independent experiments from healthy donors and shows standard error bars.

Supplementary Figure 1: MMP-1, 2, 7 and 10 are produced by human MDMs at 24h post-infection, but are not downregulated in ΔESX1

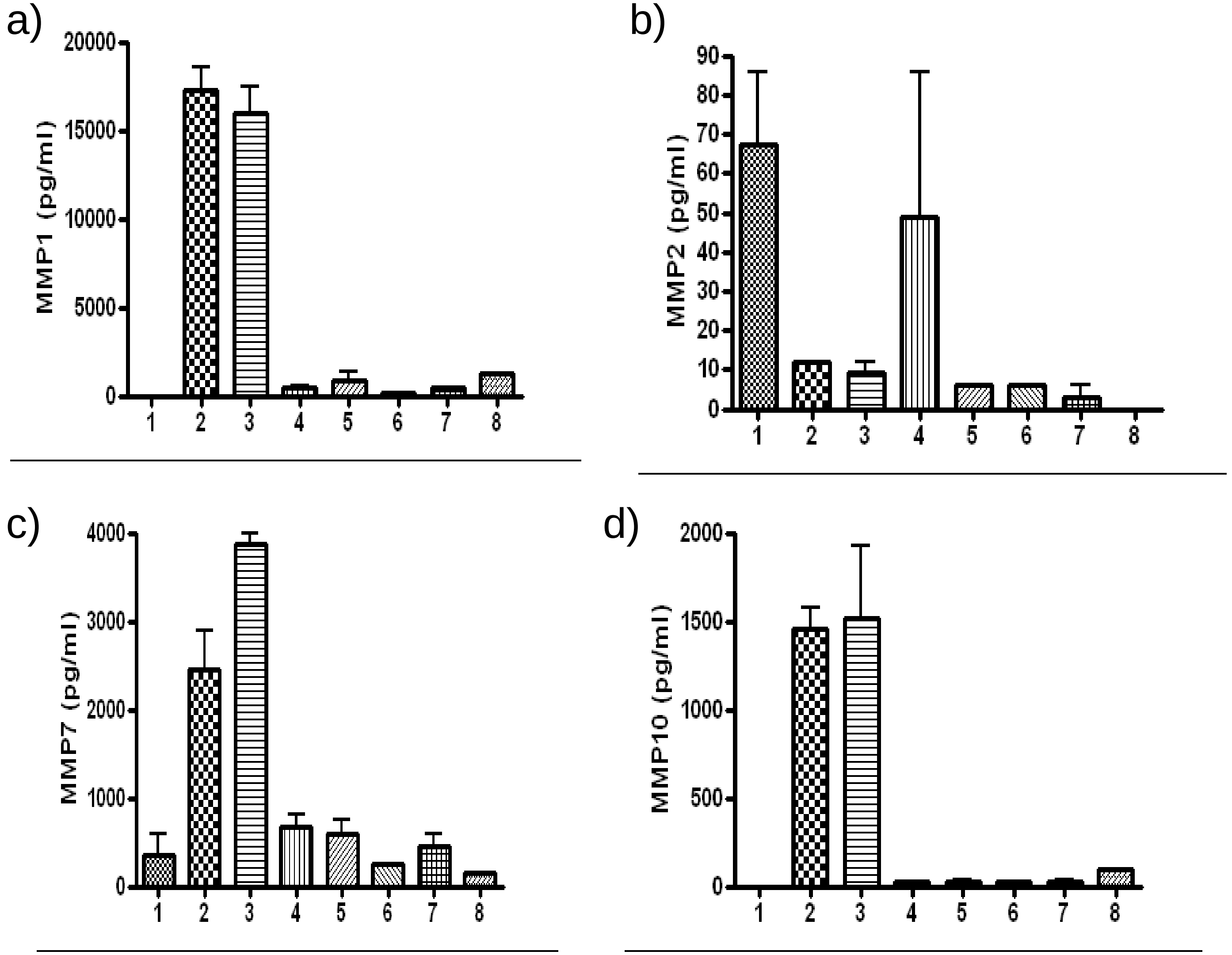

Supplement: Supplementary file 1 [file mmc1.pdf]
